# New approaches regarding the *in vitro* maturation of oocytes: manipulating cyclic nucleotides and their partners in crime

**DOI:** 10.5935/1518-0557.20170010

**Published:** 2017

**Authors:** Ramon Cesar Botigelli, Eduardo Montanari Razza, Elisa Mariano Pioltine, Marcelo Fábio Gouveia Nogueira

**Affiliations:** 1 Department of Pharmacology, Institute of Bioscience, University of São Paulo State, Botucatu, São Paulo, Brazil; 2 Department of Biological Sciences, Faculty of Sciences and Letters, University of São Paulo State, Assis, São Paulo, Brazil

**Keywords:** Oocytes, cumulus cells, oocyte *in vitro* maturation, cyclic nucleotides, *in vitro* oocyte maturation techniques

## Abstract

Several discoveries have been described recently (5-10 years) about the biology of ovarian follicles (oocyte, cumulus cells and granulosa cells), including new aspects of cellular communication, the control of oocyte maturation and the acquisition of oocyte competence for fertilization and further embryo development. These advances are nourishing assisted reproduction techniques (ART) with new possibilities, in which novel culture systems are being developed and tested to improve embryo yield and quality. This mini-review aims to describe how the recent knowledge on the physiological aspects of mammalian oocyte is reflecting as original or revisited approaches into the context of embryo production. These new insights include recent findings on the mechanisms that control oocyte maturation, especially modulating intraoocyte levels of cyclic nucleotides during *in vitro* maturation using endogenous or exogenous agents. In this mini-review we also discuss the positive and negative effects of these manipulations on the outcoming embryo

## BACKGROUND

The literature concerning oocyte competence and embryo quality has become abundant in the last few years. Several factors are involved in oocyte metabolism, cyto-skeletal remodeling, accumulation of molecules (RNAs), meiosis arrest/resumption and fertilization, all of which are key events for initiating and sustaining early embryogenesis. This large amount of published data has allowed researchers to pursue new strategies in assisted reproduction techniques (ART). This mini-review focuses on the recent progress in understanding the events controlling the acquisition of competence in oocytes and the new mechanisms involved in the maintenance of oocyte meiotic arrest; and thus, it may yield future approaches regarding the development of novel systems of *in vitro* culture, and hopefully bring the status of *in vitro* production of embryos (IVP) to a whole new level. For the purpose of this mini-review the literature search was performed in the PubMed database, to find all relevant papers focusing in "mammalian oocyte maturation", "*in vitro* oocyte maturation techniques" and crossing data with "cyclic nucleotides" ("cyclic adenosine monophosphate", "cyclic guanosine monophosphate"), "meiosis arrest/resumption" and "embryo yield", from which we have selected 112 papers, among original and review papers, to discuss the most interesting findings and also included some of our own.

## INTRODUCTION

Mammalian oocytes pass through a long and complex process to acquire the competence necessary for fertilization and embryogenesis. Oocytes are formed in fetal life, when the primordial germ cells (PGC), first seen in the epiblast, move outside the embryo to the yolk sac, and then migrate from the yolk sac to the early gonad (genital ridges). After genital ridges are colonized by PGC they are denominated primordial gonads (review by van den Hurk & Zhao, 2005).

The migration and colonization of the gonads by PGC in females give rise to the oogonia, which, associated with somatic cells, undergo a phase of mitotic proliferation with an incomplete cytokinesis. In the developing ovary, the oogonia and pregranulosa somatic cells progressively organize into epithelial structures eventually recognized as ovarian follicles. However, before follicle formation, germ cells change from mitotic to meiotic and become primary oocytes, committed to follicle development (reviewed by Guigon & Magre, 2006).

Still in fetal phase, all female germ cells reach the prophase of the first meiotic division, but instead of progressing to metaphase, they are kept arrested in the diplotene stage or germinal vesicle (GV). After birth, oocytes undergo some important processes for their growth and maturation, such as storage of mRNA, proteins, metabolic substrates and organelle reorganization. The primary oocytes, arrested in the first meiotic division, now become enveloped by a layer of flattened pregranulosa cells and a basal membrane to become primordial follicles (Picton, 2001).

Followed by the activation of growth, the primordial follicle is surrounded by a complete layer of cuboidal granulosa cells making it a primary follicle (Picton, 2001). During follicular growth, granulosa cells continue to proliferate and the theca layer is developed, which is required to produce androgens and to form the network of cells that support the vascular system of the growing follicle (Young & McNeilly, 2010).

Primordial follicles remain 'dormant' in the ovaries until recruitment into the population of growing cells. Every day, a group of primordial follicles are recruited and start to grow based on the order in which they are initially formed. Consequently, certain primordial follicles are first transformed into primary follicles after a few days and others only after more than a year as in rodents, or after one to five decades later, as in women (van den Hurk & Zhao, 2005).

Follicles are called primary follicles when the single layer of granulosa cells surrounding the oocyte becomes cuboidal. The transition of primordial follicles into primary follicles is slow and the diameter of its oocyte hardly changes. This process is associated with commitment and subsequent stages of follicular development, and it is independent of direct FSH action (Méduri *et al*., 2002).

Furthermore, when the follicle reaches several layers of granulosa cells, it starts forming the antrum, and the granulosa cells differentiate into two compartments: the mural cells, which internally surround the basal membrane, and the *cumulus oophorus* cells (CCs), that are closely associated with the oocyte. This structure forms the so-called cumulus-oocyte complex (COC) and its intricate interaction confers the oocyte with the competence to resume meiosis and be fertilized (Hyttel *et al*., 2010; Picton, 2001).

### Bidirectional communication between oocytes and somatic cells

The granulosa cells have clearly established roles to support oocyte growth and the acquisition of developmental competence (Brower & Schultz, 1982), but also participate in the control of meiosis progression (Eppig, 1991), and in the modulation of global transcriptional activity and chromatin remodeling in the oocyte (De La Fuente & Eppig, 2001).

In recent years, researchers have focused on the understanding how the oocyte can influence granulosa cells through the so-called oocyte derived paracrine factors (ODPFs). Among them, the main players are the proteins of the transforming growth factor β (TGF-β) superfamily, such as the growth differentiation factor 9 (GDF9) and the bone morphogenetic protein 15 (BMP15). Fibroblast growth factors (FGFs) are also secreted by oocytes and are reported to regulate granulosa cell development and function cooperatively with TGF-β proteins (reviewed by Emori & Sugiura, 2014).

Especially within antral follicles, ODPFs guide the differentiation and maintenance of granulosa and CCs (Eppig *et al*., 1997). In addition, ODPFs can stimulate growth and apoptosis (Gilchrist *et al.*, 2001), energy metabolism (Sugiura *et al*., 2005; Sutton *et al*., 2003; Sutton-McDowall *et al*., 2010), sterol biosynthesis (Su *et al.*, 2008) and the CCs expansion (Elvin *et al*., 1999; Varani *et al*., 2002). Thus, the oocyte can affect the functions of the CCs for their own benefit, since the oocyte is not able to produce all the substrates required for its maturation. To ensure an effective development, oocyte and cumulus/granulosa cells must communicate through a perfectly orchestrated signaling system.

One mechanisms of bidirectional communication between the CCs and oocyte is through the gap junctional communication (GJC). Gap junctions (GJ) are specialized membrane proteins occurring in points of very close contact between both cells. They consist of arrays of intercellular channels that allow direct sharing of small (less than 1 kD) molecules between the cells (Harris, 2001). Indeed, many of the molecules are known to be transferred from granulosa cells to the growing oocyte via GJC, *e.g.*, amino acids, glucose, and ribonucleotides (Eppig *et al*., 2005; Sugiura *et al*., 2005). GJ are comprised of connexins, a homologous family of more than 20 proteins. The connexin 43 (Cx43) is predominantly expressed by cumulus/granulosa cells whereas Cx37 seems to be the only connexin connecting oocyte to the granulosa cells (Juneja *et al*., 1999), and the loss of Cx37 expression is detrimental to the oocyte-granulosa communication (Simon *et al*., 1997).

Macaulay *et al.* (2014, 2016) demonstrated by confocal and transmission electron microscopy, in combination with transcript detection, that somatic cells contribute to the maternal reserves of oocytes, including mRNA and long noncoding RNA. This communication is performed by transzonal projections (TZPs). These recent discoveries refined our understanding of the small molecule transport mechanism (GJC/TZP) synthesized by cumulus cells, which are transferred into the ooplasm.

Recently, a new mechanism of cell communication within the ovarian follicle was demonstrated; this mechanism is performed by extracellular vesicles (EVs). Initially, EVs were described in ovarian follicular fluid of mares using flow cytometer and transmission electron microscopy techniques (da Silveira *et al*., 2012). These EVs are lipid bilayer structures secreted by many cell types into the extracellular fluid, serving as a vehicle for membrane and cytosolic proteins, lipids, and RNA (Raposo & Stoorvogel, 2013). Several articles identified miRNAs in bovine (Miles *et al*., 2012), equine (da Silveira *et al*., 2012) and human (Santonocito *et al*., 2014) follicular fluid, suggesting EVs as a potential mediator of cell-to-cell communication, impacting oocyte and follicle growth (reviewed by da Silveira *et al*., 2015).

### Cyclic nucleotides and maturation control

Other important molecules that also use the GJC/TZP system to move around between CCs and oocyte are the cyclic nucleotides. Among those, we should highlight the adenosine 3',5'-cyclic monophosphate (cAMP). This second messenger acts mostly in the phosphorylation of the cAMP-dependent protein kinase A (PKA), leading to the activation of various cellular pathways. The cAMP is synthesized from adenosine triphosphate (ATP) by adenylate cyclase (AC), following the dissociation of the stimulatory-G (Gs) protein from specific classes of G-protein-coupled receptors (Wright *et al*., 2015). Variation in the intraoocyte concentration of cAMP can modulate the resumption of meiosis. Optimum concentration of cAMP maintains PKA active, which inhibits the maturation-promoting factor (MPF) and keeps the oocyte arrested at the GV stage (Sirard *et al*., 1998).

Another cyclic nucleotide, the cyclic guanosine monophosphate (cGMP), also plays its role in controlling meiotic arrest/resumption. cGMP is synthesized via different pathways, such as through nitric oxide (NO), bicarbonate, natriuretic peptides (NPPA, NPPB and NPPC), guanylins, uroguanylins and guanylyl cyclase activating proteins (GCAPs); those guanylin molecules can activate various enzymes, e.g., guanyl, adenylyl cyclases and guanylate, which act in the catalytic conversion of guanosine triphosphate (GTP) into cGMP and pyrophosphate (Potter, 2011).

Like cAMP, the cGMP molecules participate in protein kinase phosphorylation (cGMP-dependent protein kinase, PKG) and influence the activity of several phosphodiesterases (PDEs). The PDEs are intracellular enzymes that catalyze the hydrolysis of the cyclic phosphate bond into cAMP and cGMP to generate the inactive products 5'-AMP and 5'-GMP (Francis *et al*., 2011). The PDEs are classified into 11 families according to their affinity, although each family can have multiple isoforms (Francis *et al*., 2011). PDE activities can be of short or long term, and are modulated by signals including hormones, neurotransmitters, cytokines, light, and oxidative influences. The concentration of nucleotides (cAMP and cGMP) is controlled by the balance between their synthesis and degradation, which is carried out by the PDEs themselves (reviewed by Francis *et al*., 2011).

PDEs decrease cAMP concentration in immature oocytes to allow for meiosis resumption and, consequently, the onset of oocyte maturation (Sadler & Maller, 1989). Tsafriri *et al.* (1996) reported that the location of PDE3A is restricted to the oocyte and they showed an effectively inhibition of spontaneous meiosis resumption *in vitro* using specific inhibitors. Additionally, Norris *et al*. (2009) demonstrated that cGMP synthesized by CCs moves across GJ/TZP to the oocyte and inhibit cAMP degradation by PDE3A. This process assures that the cAMP concentration, demanded by the GV-arrest, be maintained at optimum levels.

During the normal reproductive cycle, a surge of LH induces oocyte maturation and ovulation (Richards *et al*., 2002). Triggered by LH, a receptor coupled to G protein is activated in the theca and granulosa cells (Breen *et al*., 2013; Gudermann *et al*., 1992; Rajagopalan-Gupta *et al*., 1998), inducing a rapid reduction in follicle cGMP, which is diffused out of the oocyte through GJ/TZP (Shuhaibar *et al*., 2015). Simultaneously, LH-induced phosphorylation and activation of PDE5 leads to decreasing levels of cGMP and relieves the inhibition of PDE3A in the oocyte, lowering cAMP content and allowing meiosis to resume (Egbert *et al*., 2016).

Knowledge of the physiology involved in oocyte meiotic arrest/resumption and maturation has enabled the development and improvement of techniques for the *in vitro* maturation (IVM) of oocytes. The IVM of mammalian oocytes is an essential tool for the basic or applied aspects of assisted reproductive technology (ART) such as developmental biology, *in vitro* production (IVP) of embryos, cloning, stem cells and embryology (Smitz *et al*., 2011). However, the efficiency of IVM is still low when compared to *in vivo* maturation, which limits its application in ART (Gilchrist, 2011). Drawbacks of IVM include decreased preimplantation embryo development, low pregnancy rates and poor live birth index (Child *et al*., 2002; Eppig *et al*., 2009). This is probably caused by the inefficiency of the oocyte to avoid the drastic decrease in cAMP concentration when removed from the follicular environment during ART procedures (Luciano *et al*., 2004; Mattioli *et al*., 1994). This spontaneous resumption of oocyte meiosis causes incomplete cytoplasmic maturation, and the asynchrony between cytoplasmic and nuclear maturation, affects oocyte development and embryo quality (Blondin *et al*., 1997; Gilchrist & Thompson, 2007; Lonergan *et al*., 2003).

Several authors have reported reversible inhibition of spontaneous meiotic resumption by pharmacological methods and most of these strategies are described in the following section of this review.

### Pharmacological approaches to modulate cyclic nucleotides during *in vitro* maturation

Reversible inhibition of meiotic resumption by pharmacological methods have been long tried by many researchers, but results on the subsequent developmental competence is variable and often lower than in COCs cultured without inhibition (Fulka *et al*., 1991; Lonergan *et al.,* 1997;; Avery *et al.,* 1998; Kubelka *et al.*, 2000; Mermillod *et al.,* 2000). In addition, pharmacological manipulations may, occasionally, affect oocytes and embryos at the ultrastructural level as well (Faerge *et al.,* 2001; Lonergan *et al.,* 2003; Nogueira *et al.,* 2003, 2005; Vanhoutte *et al.,* 2007).

In mammalian oocytes, among the pharmacological approaches used for *in vitro* maturation to maintain meiotic arrest - or at least to retard meiotic spontaneous resumption - we have the cAMP modulators: dbcAMP (Sirard & First, 1988) and 8-bromo-cAMP (Chen *et al.,* 2009), phosphodiesterase inhibitors: specific inhibitors of the PDE3, such as, cilostamide (Gharibi *et al.,* 2013; Mayes & Sirard, 2002; Shu *et al.,* 2008; Vanhoutte *et al.,* 2008) and milrinone (Mayes & Sirard, 2002; Thomas *et al.,* 2002, 2004b), PDE4: rolipram (Mayes & Sirard, 2002; Thomas *et al.,* 2002, 2004b) and PDE8: dipyridamole (Sasseville *et al.,* 2009), nonspecific inhibitor: IBMX (Albuz *et al.,* 2010; Rose *et al.,* 2013; Thomas *et al.,* 2002) and stimulators of adenylate cyclase: forskolin (Albuz *et al.,* 2010; Richani *et al.,* 2014; Shu *et al.,* 2008; Zeng *et al.,* 2014) and iAC (Aktas *et al.,* 1995; Guixue *et al.,* 2001; Luciano *et al.,* 2004).

The overall objective of these pharmacological manipulations is to avoid premature nuclear maturation *in vitro* by means of maintaining higher concentration of cAMP within the ooplasm. This should provide enough time for the COC to synchronize nuclear and cytoplasmic maturation, as similar as possible to the *in vivo* event that would allegedly result in more competent oocytes and embryo (Thomas *et al.,* 2004a). Unsurprisingly, the mechanism of synthesis and hydrolysis of cGMP is one of the main targets of pharmacological strategies to control oocyte maturation.

Nakamura *et al.* (2002) described an important role of inducible nitric oxide (NO) synthase (iNOS)/NO/cGMP in the control of oocyte maturation in rats. This technique uses a NO donor (*S*-nitroso-L-acetyl penicillamine - SNAP) for 5 hours to reversibly prevent GV breakdown. This discovery paved the way for many other studies that also reported the activation of this pathway in several mammalian species, including rats (Sela-Abramovich *et al.,* 2008), mice (Norris *et al.,* 2009), pigs (Chmelíková *et al.,* 2010; Tichovská *et al.,* 2011) and bovines (Pires *et al.,* 2009; Sasseville *et al.,* 2008; Schwarz *et al.,* 2008, 2010).

New approaches for modifying IVM and improve developmental competence take into consideration the knowledge from cGMP/cAMP and the use of dynamic systems. Furthermore, non-pharmacological strategies are trending since the 2010 paper from Dr. John Eppig, describing the role of the granulosa cell ligand natriuretic peptide precursor type C (NPPC) and its receptor NPR2 in maintaining meiotic arrest in mice oocytes.

### Novel systems of *in vitro* maturation and their impacts in the resulting embryo

Based on the significant advances of the mechanisms that control oocyte maturation and their interaction with the CCs, new paths were opened to improve the IVM technique. One of which is the use of dynamic *in vitro* systems to improve embryo quality and quantity, the so-called prematuration or pre-IVM systems.

**Figure 1:**
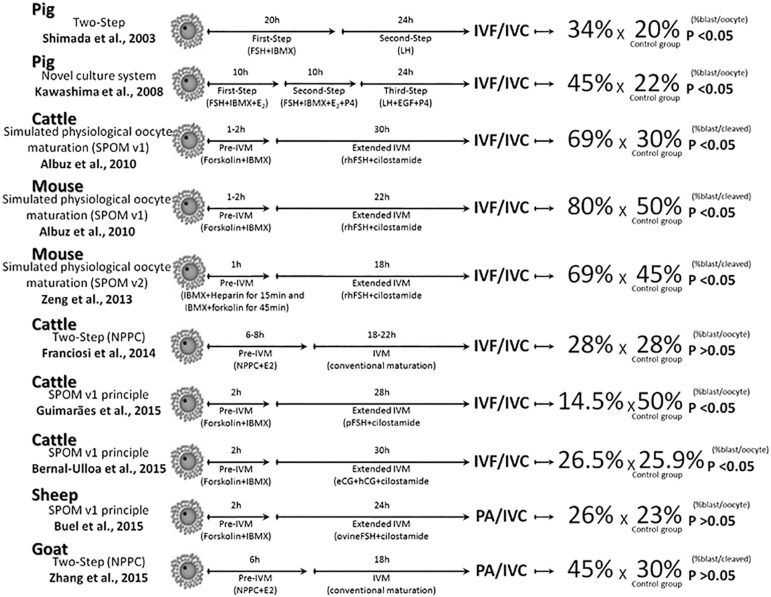
Summarized representation of different strategies used during in vitro maturation to modulate the intraoocyte concentration of cyclic nucleotides and improve embryo yield in several mammalian species (pigs, cattle, mice, sheep or goat). After each approach, there is a brief schematic description of the methods the authors used. The figure shows the rates of blastocyst in each treatment in comparison to their respective control group. Statistical significance is indicated by the p value. Note that some authors calculate the in vitro performance by dividing the number of blastocyst by the number of oocytes and others by the number of cleaved embryos. IVF: in vitro fertilization; IVC: in vitro culture; PA: parthenogenetic activation.

Interesting approaches for modifying IVM to improve developmental competence is the use of a two-step culture or pre-maturation systems, where during the initial step a medium that does not promote nuclear maturation is used (Albuz *et al.,* 2010; Franciosi *et al.,* 2014; Luciano *et al.,* 2004; Oliveira e Silva *et al.,* 2011; Ponderato *et al.,* 2002). Among the systems that have been developed, the most promising are those that pharmacologically inhibit or retard meiotic resumption by elevating cAMP concentration in the oocyte while sustaining GJ communication functionality (Albuz *et al.,* 2010; Luciano *et al.,* 2004). It was previously reported that modulation of cAMP levels within mammalian COCs during IVM could substantially improve oocyte developmental competence in several species (Albuz *et al.,* 2010; Funahashi *et al.,* 1997; Luciano *et al.,* 1999, 2004; Nogueira *et al.,* 2003, 2006; Shu *et al.,* 2008; Thomas *et al.,* 2004b; Vanhoutte *et al.,* 2009; Zeng *et al.,* 2013; Figure 1).

In 2003, Shimada *et al.* (2003) investigated the formation of LH receptor in cumulus cells of swine COCs and, after its detection, a new two-step culture system was developed. In the first step, the COCs were cultured in medium supplemented with FSH and IBMX for 20h, followed by culture in medium supplemented with LH (second-step). This two-step system with FSH and 0.5 mM IBMX induced the expression of LH receptors in CCs, improved the rate of blastocyst formation and increased the number of cells in IVF blastocysts (Figure 1).

To better understand the influence of hormones and growth factor production on the mechanisms controlling the *in vivo* maturation in pigs, Kawashima *et al.* (2008) updated the two-step system into a new one called "novel culture system - NCS". In the NCS design, COCs are recovered from small antral follicles (3-5mm in diameter); first pre-IVM uses FSH, E2 and IBMX for 10h to induce cell proliferation; second pre-IVM takes place with FSH, E2, IBMX and P4 for 10h to suppress cell proliferation and induce LH receptor mRNA expression and finally, an IVM with LH, EGF and P4 for additional 24h (Figure 1). Using NCS system, Kawashima *et al.* (2008) reported the full expansion of porcine COCs, decreased number of cumulus cells in apoptotic process and, when oocytes obtained from NCS were used for IVF, the developmental competence to blastocyst was significantly improved when compared with the conventional culture system (FSH+LH for 48h), or with the two-step culture system (Funahashi *et al.,* 1997; Shimada *et al.,* 2003; Figure 1).

Early in the decade, Albuz *et al.* (2010) proposed a new IVM system. Their methodology was seeking to mimic the processes that occurred in the *in vivo* maturation; hence their system was called simulated physiological oocyte maturation (SPOM; Figure 1). This system consisted of a small pre-IVM (1-2h) where the adenylate cyclase was stimulated with forskolin, increasing cAMP levels and IBMX, a PDE inhibitor, to prevent hydrolysis of cyclic nucleotides (cAMP and cGMP), and after the pre-IVM, the COCs were subjected to an extended IVM for 24 hours, where the culture medium was supplemented with cilostamide (PDE3 inhibitor) and recombinant human (rh)-FSH. Results of the SPOM system were quite exciting, with rates of 69% of blastocysts per cleaved embryo. Later, the SPOM protocol was adapted to sheep oocytes and, even though no significant effect on blastocyst rates were achieved, there was an improvement in blastocyst quality observed by an increase in total cell number (Rose *et al.,* 2013; Figure 1).

The promising results of the first version of the SPOM system (SPOMv1) greatly impacted ART research; still, the SPOM system was updated by their creators in subsequent studies. Zeng *et al.* (2013) used heparin during pre-MIV and removed the cilostamide from the extended IVM. This approach positively affected oocyte energy metabolism, oocyte meiotic maturation and embryo development (SPOMv2; Figure 1).

One year later, Zeng *et al.* (2014) tested the presence of rh-FSH during extended IVM (Figure 1). By now it seems that cilostamide in extended IVM phase is gone for good, and now the system is no longer called SPOM, but Prematuration System (or Pre-IVM system) instead. They reported successful results in IVP of mice embryos (± 70% of blastocysts per cleaved embryos), better quality in the expansion of CCs, reduction of abnormal spindles and a positive influence of Pre-IVM system in the glycolic metabolism of COCs (suggesting an effect of cAMP production predominantly on glycolytic activity).

Several laboratories and research groups around the world sought to repeat the success obtained by the SPOM or Pre-IVM system; however, most failed in doing so (Bernal-Ulloa *et al.,* 2016; Buell *et al.,* 2015; Guimarães *et al.,* 2015; Razza *et al.,* 2015; Figure 1). Ulloa *et al.* (2015), using bovine oocytes cultured in SPOM system, produced a smaller number of blastocyst compared with the standard IVM. However, the pattern of DNA methylation of embryos produced in SPOM system was more similar to embryos produced *in vivo.* Also, Santiquet *et al.* (2014a) tested a pre-IVM treatment and could improve the developmental competence of oocytes, as demonstrated by increased embryo development. Additionally, pre-IVM performed with IBMX and forskolin in the Pre-IVM system can change ultrastructural characteristics of oocytes and blastocysts (Razza *et al.,* 2015; unpublished data from our group).

With the strategy used in the SPOM system of performing a two-step culture with different drugs to induce different effects in CCs and oocytes during IVM, new drugs and signaling pathways have emerged as potential targets for research seeking to improve IVM and embryo production.

Earlier in this decade, a new model discovered that the binding of NPPC to its receptor (NPR2) in granulosa and CCs are the main cause for the modulation of cGMP levels (Zhang *et al.,* 2010). Until now, studies relating the new mechanism of NPPC/NPR2/cGMP in maturation control have been reported in mice (Tsuji *et al.,* 2012; Zhang *et al.,* 2011), bovines (Franciosi *et al.,* 2014), swine (Santiquet *et al.,* 2014b; Zhang *et al.,* 2014) and sheep (Zhang *et al.,* 2015; Figure 1).

Using the NPPC during pre-IVM (8h) in bovine COCs, Franciosi *et al.* (2014) could successfully arrest meiosis resumption and extend the functional communication among oocytes and CCs through GJ. After IVF and embryo culture, the NPPC treatment in pre-IVM has also increased blastocyst cell number and hatching rates.

In a more recent approach of two-step maturation with caprine COCs, Zhang *et al.* (2015) used the NPPC and estradiol during pre-IVM (8h), followed by conventional IVM (18h). With this system, meiosis was effectively arrested in pre-IVM and the maturation rate was also increased after conventional IVM. They also increased embryo production and quality, evaluated by total cell number per blastocyst (Figure 1).

Conventionally, oocyte competence has been assessed by embryo morphology and blastocyst rates; however, these aspects alone do not provide sufficient information to fully endorse the IVM system efficiency. Several strategies are being used to study the quality of oocytes (before and after IVM) and embryos. Some of the most promising and approaches that focus on the identification of biomarkers.

### New perspectives and final considerations

A few non-invasive strategies are already being used to predict oocyte competence to become a viable embryo, even before the oocyte is fertilized. These approaches aim to identify oocyte competence biomarkers mostly in cumulus cells. In this context, the morphology of CCs can be used to first categorize oocyte potential (de Loos *et al.,* 1991) and then to compare the morphological data with CCs transcriptome (differentially expressed genes in CCs surrounding the good oocytes versus poor-quality oocytes). At present, many genes are identified as potential biomarkers (reviewed by Labrecque & Sirard, 2014). Still, many research groups are working on the identification of miRNAs as biomarkers as well. Profiles of miRNAs isolated from EVs present in follicular fluid were described and associated with proper cytoplasmic oocyte maturation; hence, these miRNA profiles can be used to predict oocyte competence (Sohel *et al.,* 2013).

The use of non-invasive strategies, such as analysis of follicular fluid and culture media (after culture) also appears to be quite useful on the search for molecular biomarkers for oocyte competence. The presence of cytokines and growth factors in follicular fluid is crucial for determining oocyte quality (reviewed by Dumesic *et al.,* 2015). In this context, the metabolic characterization of the culture media, in which IVP embryos are kept for many hours, may represent an important non-invasive tool to either indicate possible predictive biomarkers of viability or to explain IVP outcome afterwards (Muñoz *et al.,* 2014).

Lipid metabolism is induced in COCs during oocyte maturation and contributes to oocyte and embryo development (Gardner & Harvey, 2015). Specific fatty acids have distinct effects on oocyte maturation. In general, saturated fatty acids (palmitic acid and stearic acid) are elevated in follicular fluid and, in some metabolic contexts, are detrimental, while the presence of unsaturated non-esterified fatty acids (oleic acid and linoleic acid) can counteract these detrimental effects and promote developmental competence (reviwed by Dunning *et al.,* 2014). Thus, *in vitro* oocyte and embryo development may be optimized through the provision of appropriate energy substrates and essential co-factors during ART in domestic animals and subfertility women.

Despite the large number of publications in the field we still have a long way to go to deeply understand and manipulate the mechanisms controlling oocyte maturation. Overcoming these gaps may allow us to improve ART results. Therefore, it is necessary to design studies aiming at finding effective biomarkers for oocyte competence. The field of the OMICs seems to be quite promising, especially regarding the new findings in transcriptomics, proteomics and lipidomics in oocytes, CCs, embryos and in the EVs within the follicular fluid. Future studies on this subject might enable the design of more complex, defined and efficient culture conditions for oocytes to be fully matured and able to generate optimum IVP embryos.
